# Expansion Speed as a Generic Measure of Spread for Alien Species

**DOI:** 10.1007/s10441-019-09366-8

**Published:** 2019-09-28

**Authors:** Hanno Sandvik

**Affiliations:** 1 Centre for Biodiversity Dynamics (CBD), Norwegian University of Science and Technology (NTNU), 7491 Trondheim, Norway; 2 Present Address: Norwegian Institute for Nature Research (NINA), 7485 Trondheim, Norway

**Keywords:** Area of occupancy, Biological invasion, Detection rate, Dispersal velocity, Grid occupancy, Invasiveness

## Abstract

The ecological impact of alien species is a function of the area colonised. Impact assessments of alien species are thus incomplete unless they take the spatial component of invasion processes into account. This paper describes a measure, termed expansion speed, that quantifies the speed with which a species increases its spatial presence in an assessment area. It is based on the area of occupancy (AOO) and can be estimated from grid occupancies. Expansion speed is defined as the yearly increase in the radius of a coherent circle having the same area as the AOO, irrespective of whether the increase is due to natural dispersal or anthropogenic transport. Two methods for estimating expansion speed are presented: one that requires several years of spatio-temporal observation data and explicitly takes detection rates into account; and one that can be used under a situation with sparse data. Using simulations and real-world data from natural history collections, it is shown that the method provides a good fit to observational datasets. Expansion speed has several valuable properties. Being based on AOO, it is an intuitive measure; as it only requires occupancy data, it is comparatively easy to estimate; and because it is a quantitative and generic measure, it increases the testability and comparability of impact assessments of alien species.

**Electronic supplementary material:**

The online version of this article (10.1007/s10441-019-09366-8) contains supplementary material, which is available to authorized users.

## Introduction

Alien species spreading into novel environments have long been recognised as a major threat to biodiversity (e.g. Pimentel 2011; Pyšek et al. 2012; Lockwood et al. 2013; Bellard et al. 2016). The ecological impact of an alien species can be defined as the product of (1) the area colonised, (2) the density attained on this area, and (3) the per-capita effects exerted on native biota or abiotic conditions (Parker et al. 1999), where the latter two factors can be combined as (per-locality) ecological effect (Sandvik et al. 2019). A variety of assessment methods exists that classify or quantify the risk posed or the impact exerted by alien species on native biota (e.g. Baker et al. 2008; Essl et al. 2011; Blackburn et al. 2014; D’hont et al. 2015; Pergl et al. 2016; Sandvik et al. 2019; for an overview, see Verbrugge et al. 2010; Roy et al. 2018). While most of these risk assessment schemes emphasise the ecological effect dimension, the spatial dimension of an invasion process is also important. Because this spatial dimension is dynamic (i.e. the area colonised increases during an invasion process) and prioritisation of management efforts often needs to identify the species that have the greatest *increase* in impact, one needs a measure of the increase in the area colonised.

This paper suggests expansion speed as a quantitative measure of this spatial dimension and presents a mathematical model that allows its estimation. Expansion speed is intended to capture the ability of a species to increase its spatial presence in an assessment area (e.g. a country). In order not to confound area with density, spatial presence must be measured on a sufficiently large scale, so that it remains independent of the ecological effect of the species.

The suggestion outlined here is to base this measure on the area of occupancy (AOO, the cumulative area of all occupied grid cells; IUCN 2017, pp. 48–49). Alongside the extent of occurrence (EOO, the smallest convex polygon encompassing all occurrences; IUCN 2017, pp. 46–48), AOO is by now established as a central parameter in ecology, biogeography and conservation biology (Gaston 1991, 1994; Quinn et al. 1996; Breiner and Bergamini 2018). For instance, it enters the IUCN Red List criteria (IUCN 2012) both in terms of its absolute size (criteria B2 and D2) and its decline (subcriteria Ac, Bb). AOO can be used for the quantification of the invasiveness of alien species, too (Wilson et al. 2014; McGeoch and Latombe 2016).

Statistical models of biological invasions have a long history (e.g. Fisher 1937; Skellam 1951; van den Bosch et al. 1990; Kot et al. 1996; Tufto et al. 1997; Petrovskii and Li 2006). These models are concerned with describing the spatial and temporal behaviour of an invasion front. More recently, considerable progress has been made with deterministic models, which describe short-distance and long-distance dispersal in terms of the underlying morphological traits of propagules and the fluid-dynamical properties of the atmospheric boundary layer (e.g. Nathan et al. 2002, 2003; Trakhtenbrot et al. 2005, 2014; Skarpaas and Shea 2007; Caplat et al. 2012). A third area of research concerns the modelling and prediction of range expansion by means of species distribution models (e.g. Dullinger et al. 2009; Veran et al. 2016; Barbet-Massin et al. 2018; Lins et al. 2018; Sullivan and Franco 2018).

Expansion speed, as suggested here, has a different motivation. It is a descriptive measure meant to quantify the *actual* temporal change in the spatial presence of a species *irrespective* of the mechanisms involved. Expansion speed is thus not to be understood as an attempt to measure dispersal velocity, nor as an attempt to predict future range expansion. This choice is deliberate, as many alien species increase rapidly in their spatial presence without having a classical invasion front. Due to anthropogenic transport of individuals, intentional and otherwise, alien species may expand even though their ranges (their EOOs) remain stable, and even though their natural dispersal velocities are low. After giving a formal definition of expansion speed, this paper presents a model to estimate this measure from data on grid occupancies, and tests the model using simulations and real data on twelve alien plant species.

## Definition

*Expansion* is here defined as the increase in the number of occurrences, where *occurrence* refers to a grid occupancy, i.e. a grid cell of specified size (e.g. 4 km^2^) occupied by the species. The number of occurrences is thus directly proportional to the AOO, as defined by the IUCN Red List criteria (IUCN 2017, pp. 48–49). In accordance with IUCN’s guidelines, AOO is understood as the specific area that is inhabited by a species, excluding cases of vagrancy. In the case of sessile organisms, therefore, any grid cell in which a viable individual is observed, necessarily counts as an occurrence.

*Expansion speed* is defined as the annual increase in the radius of AOO, where “radius of AOO” is understood as $\sqrt {{\text{AOO}} \cdot \pi^{ - 1} }$. This means that the radius is calculated *as if* the AOO was a coherent circle containing all occurrences and only occurrences, irrespective of whether this is the case (in reality, of course, such an assumption would never be met). Because the AOO is just the number of grid occupancies multiplied by the area of one grid cell, AOO is independent of where the occupied grid cells are situated. Therefore, a coherent or circular AOO is not a *prerequisite* for measuring expansion in terms of increase in radius—to the contrary, expansion is indifferent to the distance and spatial distribution of occurrences. Rather, “increase in the radius of AOO” can be considered as a way of *standardising* the measure and illustrating its meaning. Expansion speed can thus be envisaged as the yearly extension in all directions of a coherent circle having the same area as the AOO. Therefore, the measurement unit of expansion speed is metres per year (m/a).

Each new occurrence contributes to expansion, irrespective of the mechanisms or pathways involved. This means that a species may expand due to its natural dispersal modes (active or passive, short-distance or long-distance), due to human transport (intentional or unintentional) or any combination of these.

Expansion speed may vary over time, e.g. due to lag phases or saturation. In such cases, the *highest realistic* value of expansion speed that is measured, estimated or reported should be used. The rationale for this recommendation is that expansion potential should be regarded as a propensity of a given species in a given environment, and conditions that temporarily slow down expansion should thus be disregarded (see Sect. 6.3 for elaboration).

In contradistinction to other invasion models that use similar approaches (e.g. van den Bosch et al. 1990; cf. Lensink 1997), the definition presented here does not require the existence of an invasion front. Earlier, Hill et al. (2001) have measured “velocity of range expansion” in the way suggested here, using occupancy of 5 km × 5 km grid cells, albeit without a formal model and without addressing detectability (see also Ward 2005; Widenfalk et al. 2014; Wilson et al. 2014; McGeoch and Latombe 2016).

## Estimation

### Modelling Expansion Speed and Detectability

Expansion speed may be estimated based on a set of specific assumptions, which are realistic for many (but admittedly not all) expansion processes of alien species, and some of which will be relaxed in Sect. 3.4. These assumptions are (a list of all variables used can be found in Table 1):

**Table 1:** Abbreviations of variables used in the equations and the text

Parameter	Description	Definition
*A*	AOO (area of occupancy) in a given year	*A*_*t*_ = *α*_*t*_ · *A*_0_
*A*_0_	Area of one grid cell (i.e. the AOO at *t* = *t*_0_)	
*b*	Slope (only used in Fig. 1)	
*K*	The maximum colonisable AOO	
*n*	Length of the time series (from *t*_0_ to *z* inclusive)	
*p*	Detection rate (strictly, detection probability); proportion of previously undetected occurrences that are detected within a given year	
*r*	Radius of the real or *total* AOO	$r_{t} = \sqrt {\alpha_{t} \cdot A_{0} \cdot \pi^{ - 1} }$
*r*_0_	Radius of the area of one grid cell	$r_{0} = \sqrt {A_{0} \cdot \pi^{ - 1} }$
*t*	Time (year)	
*t*_0_	Time (year) at which expansion starts with the colonisation of the first grid cell	
*U*	Uniformly distributed random variable within the bounds ]0;1[	
*v*	Expansion speed	$v = \Delta r \cdot \Delta^{ - 1} t$
*z*	Last year in the time series of observations	
*α*	Total number of occurrences (detected and undetected) in a given year	
*γ*	Annual multiplication rate of occupied grid cells	$\gamma = \Delta \ln \alpha \cdot \Delta^{ - 1} t$
*δ*	“Detection debt” (time span by which the asymptote of ρ is displaced relative to the regression line of *r*; cf. Fig. 1)	$\delta = p^{ - 1} - 1$
Δ	Difference (only used in this table)	$\Delta x = x_{2} - x_{1}$
*η*	Sampling effort in a given year	
*κ*	Proportionality factor between sampling effort and detection rate	$\kappa = p \cdot \eta^{ - 1}$
*λ*	Average number of new grid cells colonised each year	$\lambda = \Delta \alpha \cdot \Delta^{ - 1} t$
*ξ*	“Dark figure”; factor by which the known AOO has to be multiplied in order to obtain the total AOO	$\xi_{t} = \alpha_{t} \cdot \varOmega_{t}^{ - 1}$
*π*	A circle’s circumference : diameter ratio	$\pi \approx 3.14159$
*ρ*	Radius of the *known* AOO	$\rho_{t} = \sqrt {\varOmega_{t} \cdot A_{0} \cdot \pi^{ - 1} }$
*τ*	Time span between two AOO estimates	
*φ*	Number of occurrences yet undetected in a given year	$\varphi_{t} = \alpha_{t} - \varOmega_{t - 1}$
*ω*	Number of occurrences detected in a given year	$\omega_{t} = p_{t} \cdot \varphi_{t}$
*Ω*	Cumulative number of occurrences that have been detected up to and including a given year	$\varOmega_{t} = \sum\nolimits_{{t_{0} }}^{t} {\omega_{i} }$

- The area in which the species expands is divided into non-overlapping and adjacent grid cells of equal area *A*_0_, which are either colonised or not colonised by the species. A colonised grid cell is here regarded as an occurrence. In accordance with IUCN (2017), but without loss of generality, grid cells are assumed to be squares with 2 km edge length (i.e. *A*_0_ = 4 km^2^).
- Grid cells that have been colonised, remain colonised, i.e. occurrences do not disappear once established.
- The model is a discrete-time model, i.e. all times *t* are considered to be integers (here interpreted as years, although other time steps are equally possible).
- The expansion process starts at *t* = *t*_0_ with the colonisation of the first grid cell. (The value of *t*_0_ is usually unknown and will be estimated from the model. This means that *t*_0_ does not have to be equal 0 but may be e.g. 1970.)
- The likelihoods of detecting occurrences in a given year *t* are identical across occurrences and independent of the presences and detections of other occurrences.

It follows from these assumptions that the number *ω*_*t*_ of occurrences detected in a given year *t* is directly proportional, by means of the detection probability *p*_*t*_ for year *t*, to the number *φ*_*t*_ of occurrences yet undetected at the beginning of year t:

1 $$\omega_{t} = p_{t} \cdot \varphi_{t} ,$$

with

2 $$\varphi_{t} = \alpha_{t} - \varOmega_{t - 1} ,$$

where *α*_*t*_ is the total number of occurrences (detected and undetected) in year *t*, and *Ω*_*t*_ is the cumulative number of occurrences that have been detected up to and including year *t*,

3 $$\varOmega_{t} = \sum\limits_{{i = t_{0} }}^{t} {\omega_{i} } .$$

Although strictly a unitless probability or proportion, *p*_*t*_ will hereafter be referred to as detection rate. This is justified by the fact that it is defined as the proportion of undetected occurrences that are detected within one time step (year).

Because the expansion process starts at *t* = *t*_0_ with one colonised grid cell, it follows (a) that *α*_*t*_ = *φ*_*t*_ = *ω*_*t*_ = *Ω*_*t*_ = 0 for all *t* < *t*_0_, and (b) that *α*_*t*_ = *φ*_*t*_ = 1 and *ω*_*t*_ = *Ω*_*t*_ = *p*_*t*_ for *t* = *t*_0_. Inserting Eqs. 1 and 2 in 3, one thus gets

4 $$\begin{aligned} \varOmega_{t} & = \omega_{t} + \varOmega_{t - 1} \\ & = p_{t} \left( {\alpha_{t} - \varOmega_{t - 1} } \right) + \varOmega_{t - 1} \\ & = p_{t} \alpha_{t} + \varOmega_{t - 1} \left( {1 - p_{t} } \right) \\ & = p_{t} \alpha_{t} + p_{t - 1} \alpha_{t - 1} \left( {1 - p_{t} } \right) + \varOmega_{t - 2} \left( {1 - p_{t - 1} } \right)\left( {1 - p_{t} } \right) \\ & = p_{t} \alpha_{t} + p_{t - 1} \alpha_{t - 1} \left( {1 - p_{t} } \right) + \cdots + p_{{t_{0} }} \alpha_{{t_{0} }} \cdot \left( {1 - p_{{t_{0} + 1}} } \right) \cdot \left( {1 - p_{{t_{0} + 2}} } \right) \cdot \ldots \cdot \left( {1 - p_{t} } \right) \\ & = \sum\limits_{{i = t_{0} }}^{t} {p_{i} \alpha_{i} \prod\limits_{j = i + 1}^{t} {\left( {1 - p_{j} } \right)} } \, .\\ \end{aligned}$$

Now, in addition to *t*_0_, Eq. 4 has two unknowns (*p*_*t*_ and *α*_*t*_) for each known (*Ω*_*t*_) and, therefore, needs some simplifying assumptions for estimation to be feasible. Two obvious simplifications (according to which *ω*_*t*_ is treated as an expected rather than an observed value, see Sect. 3.3) are the assumption of a constant detection rate *p*,

5 $$\varOmega_{t} = p\sum\limits_{{i = t_{0} }}^{t} {\alpha_{i} \left( {1 - p} \right)^{t - i} } ,$$

or of a constant expansion speed *v*,

6 $$\varOmega_{t} = \sum\limits_{{i = t_{0} }}^{t} {p_{i} \left[ {v\left( {i - t_{0} } \right)\sqrt {\tfrac{\pi }{{A_{0} }}} + 1} \right]^{2} \prod\limits_{j = i + 1}^{t} {\left( {1 - p_{j} } \right)} } .$$

The latter is obtained by solving for *α*_*t*_ and inserting into Eq. 4 the following definition of a constant expansion speed:

7 $$v = \frac{{r_{t} - r_{0} }}{{t - t_{0} }} = \frac{{\sqrt {A_{t} } - \sqrt {A_{0} } }}{{\sqrt \pi \left( {t - t_{0} } \right)}} = \sqrt {\frac{{A_{ 0} }}{\pi }} \cdot \frac{{\sqrt {\alpha_{t} } - 1}}{{t - t_{0} }},$$

where *A*_0_ is the area of one occurrence; *A*_*t*_ = *α*_*t*_ · *A*_0_ is the AOO in year *t*; *r*_0_ is the radius of *A*_0_; and *r*_*t*_ is the radius of *A*_*t*_ (imposing a circular shape). Combining the two abovementioned simplifying assumptions (both detection rate and expansion speed are constant), one is left with three unknowns, viz. *p*, *v* and *t*_0_:

8 $$\varOmega_{t} = p\sum\limits_{{i = t_{0} }}^{t} {\left[ {v\left( {i - t_{0} } \right)\sqrt {\tfrac{\pi }{{A_{0} }}} + 1} \right]^{2} \mathop {\left( {1 - p} \right)}\nolimits^{t - i} } .$$

### Graphical Representation

**Fig. 1:**
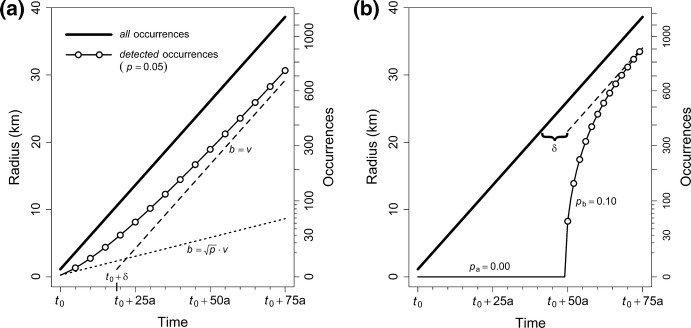
Trajectories of expansion and detection of an invading species according to the model proposed in the text, illustrated for the case of a constant expansion speed *v* = 500 m/a. In this situation, the radius *r* of the *total* AOO increases linearly (all occurrences, bold lines), whereas the radius *ρ* of the *known* AOO lags behind (cumulative detected occurrences, thin solid lines). The dotted line (**a**) shows the initial slope *b* of the detection curve; the broken lines show the asymptotes that are approached by the detection curves. The ‘detection debt’ *δ* is the time by which the asymptote is displaced from the expansion trajectory. The slopes of the true expansion trajectory and of the asymptote equal the expansion speed (*b* = *v*). The detection rate *p* is constant at 5% in (**a**), whereas it changes after 50 years from 0 to 10% in (**b**). Note that the right *y*-axes are on square-root scale (e.g. a radius *r* of 20 km corresponds to an AOO of *πr*^2^ = 1256 km^2^, and thus to 314 occurrences or grid occupancies of 4 km^2^ each)

What does the temporal change in *Ω*_*t*_, i.e. the graph of the function described by Eq. 4, look like? It is easier to visualise the *radius* of the known AOO than *Ω*_*t*_ itself. In Fig. 1, the radii of the *real or total* AOOs ($r_{t} = \sqrt {\alpha_{t} \cdot A_{0} \cdot \pi^{ - 1} }$) are shown with bold lines, the radii of the *known* AOOs ($\rho_{t} = \sqrt {\varOmega_{t} \cdot A_{0} \cdot \pi^{ - 1} }$) with thin solid lines and open circles.

The situation with a constant *v* = 500 m/a and constant *p* = 0.05 (i.e. the special case described by Eq. 8) is shown in Fig. 1a. Although it may be somewhat counterintuitive that the increase of the radius of known AOO is not linear under a constant *v* and *p*, this follows from the fact there are few occurrences to begin with. When unobserved occurrences have accumulated over several years, however, the slope of the curve will become increasingly steeper. More specifically, the curve of the radius of known AOO starts out with a slope equalling $\sqrt p \cdot v$ (proof provided in the Appendix) and asymptotically approaches a line that is parallel to the radius of the real AOO, i.e. the asymptote has slope *v* and is displaced to the right by a time span of *δ* = (*p*^−1^ – 1) years (proof provided by F.J.A. Jacobs in Online Resource 1), which might be called the *detection debt*.

The ratio between the total number of occurrences and the known number of occurrences can be referred to as the *dark figure* of the AOO, $\xi_{t} = \alpha_{t} \cdot \varOmega_{t}^{ - 1}$. For instance, a dark figure of 10 means that one tenth of the real occurrences is known. Since the number of real occurrences is unknown, the exact value of the dark figure is necessarily unknown, too. Under the assumptions of the model, the dark figure starts out as $\xi_{t} = p_{t}^{ - 1}$ at *t* = *t*_0_ and then decreases, slowly approaching 1. For example, in Fig. 1a, the dark figure is 20 in the first year, has dropped below 5 in the 11th year and below 2 in the 47th year. In the final (75th) year, 738 out of 1172 occurrences have been observed, so that the dark figure is just below 1.6.

### Maximum Likelihood Estimation

In reality, the detection rate *p*_*t*_ is neither known nor estimable for all years. Instead, one will have to assume an average detection rate $\bar{p}$. The number of occurrences detected in a given year is then binomially distributed, *ω* ~ *B*(*φ*, $\bar{p}$), so that Eq. 1 describes the *expectation* of the number of observed occurrences, *E*(*ω*_*t*_), rather than its real value. With these simplifications, it is possible to obtain maximum-likelihood estimates of *α* and *p* (were estimates are denoted using the hat operator, i.e. $\hat{\alpha }$ and $\hat{p}$) by maximising the log-likelihood function

9 $$ \begin{aligned} \ln {\mathcal{L}}\left( {{\hat{\mathbf{\alpha }}},{\hat{\mathbf{p}}}|{\varvec{\upomega}}} \right) & = \ln \prod\limits_{i = 1}^{n} {P\left( {\omega_{i} |\hat{\alpha }_{i} ,\hat{p}_{i} } \right)} \\ & = \sum\limits_{i = 1}^{n} {\left[ {\ln \left( {\begin{array}{*{20}c} {\hat{\alpha }_{i} - \varOmega_{i - 1} } \\ {\omega_{i} } \\ \end{array} } \right) + \omega_{i} \ln \hat{p}_{i} + \left( {\hat{\alpha }_{i} - \varOmega_{i} } \right)\ln \left( {1 - \hat{p}_{i} } \right)} \right]} , \\ \end{aligned} $$

where *n* is the length of the time series; and the bold variables ${\hat{\mathbf{\alpha }}}$, ${\hat{\mathbf{p}}}$ and **ω** are vectors of length *n* containing the estimated or observed values of $\hat{\alpha }_{t}$, $\hat{p}_{t}$ and ${{\omega}_{t}}$ for each year, respectively.

An alternative method to estimate the parameters is using least squares, i.e. by minimising the squared differences between the observed **Ω** and the ${\hat{\mathbf{\varOmega }}}$ predicted from Eq. 4. This corresponds to a maximum-likelihood optimisation under the assumption of normally distributed residuals. Although the latter assumption may not strictly be fulfilled, this optimisation can be more efficient in estimating the model parameters.

A script written in **R** (**R** Core Team 2017), ‘expansion’, is available for deriving maximum-likelihood or least-squares estimates of *v*, *p* and *t*_0_, given spatio-temporal data of observations (Sandvik 2017). This script converts observations into occurrences based on a standardised 2-km grid (SSB2KM; Strand and Bloch 2009); provides a summary of the input dataset (number of observations and occurrences; range of years, latitudes and longitudes); calculates the (known) EOO; may print the occurrences on a map; models the expansion process; plots the fitted detection curve; and produces an output including the parameters estimated with confidence intervals, the dark figure for the last year, the variance explained (*R*^2^) and Akaike’s Information Criterion (corrected for small sample size, AIC_C_; Hurvich and Tsai 1989; Burnham and Anderson 2002). The default model fitted to the data is the one in which both detection rate and expansion speed are constant (Eq. 8).

### Model Modifications

A number of model modifications are possible, both as relaxations of the initial assumptions or as alternative simplifications. A non-exhaustive list of such modifications includes:

1. The detection rate *p* varies proportionally to some specified frequencies. As Skarpaas and Stabbetorp (2011) have shown, it may be a good approximation to assume that detection rates for a given species are proportional to the sampling effort for a larger taxon (“background group” sensu Ponder et al. 2001). Sampling effort can in its turn be inferred from, e.g., natural history collections in museums (Delisle et al. 2003). Detection rates would then take the form *p*_*t*_ = *κ* · *η*_*t*_, where *κ* is the constant proportionality factor estimated from the data; and *η*_*t*_ is the pre-specified sampling effort in year *t*. Under this modification, *p*_*t*_ in Eq. 4 (or Eq. 6) is simply replaced by *κ η*_*t*_.
2. The detection rate *p* is allowed to change once. This is an intermediate scenario between assuming a constant *p* and a time-dependent *p*, which would imply that sampling effort underwent one major change, e.g. due to increasing awareness of the species. This can be done by inferring a breakpoint *t*_×_ from the data so that *p*_*t*_ = *p*_a_ for all *t* < *t*_×_, and *p*_*t*_ = *p*_b_ for all *t* ≥ *t*_×_. Figure 1b illustrates this modification with the extreme case of *p*_a_ = 0 (*p*_b_ = 0.1, *v* = 500 m/a).
3. The detection rate *p* varies in space. Detection rates may be higher in areas with a high human population density (Dodd et al. 2016). It may also be the case that detection rates in adjacent grid cells are not statistically independent, e.g. when sampling effort is increased around known occurrences. Such modifications can be modelled by expressing *p* as some function of human population density and/or of the detection status in neighbouring cells.
4. Expansion speed *v* drops to zero once a maximum AOO is colonised. This scenario may be preferable when the alien species is constrained to certain habitats or climatic zones, and especially when the expansion process has been going on for a long time. Different functions for *v*_*t*_ (and thus *α*_*t*_) are conceivable, the simplest of which would be a break point at which *v* instantaneously drops to zero. More realistic models would assume some kind of decelerating function, so that *α* approaches its maximum value asymptotically. This can be done by using the logistic function, e.g.
  10 $$\alpha_{t} = K\frac{{1 - e^{{{{ - 2v\left( {t - t_{0} } \right)} \mathord{\left/ {\vphantom {{ - 2v\left( {t - t_{0} } \right)} K}} \right. \kern-0pt} K}}} }}{{1 + e^{{{{ - 2v\left( {t - t_{0} } \right)} \mathord{\left/ {\vphantom {{ - 2v\left( {t - t_{0} } \right)} K}} \right. \kern-0pt} K}}} }},$$
  where *K* is the maximum AOO colonised. *K* would either have to be estimated from the data alongside *v*, *p* and *t*_0_, or it may alternatively be pre-specified based on knowledge about the total colonisable area.
5. The expansion process is characterised by an initial lag phase. Such lag phases can have a number of reasons (Crooks 2005; Aikio et al. 2010; Coutts et al. 2018). If the reason is related to detectability (i.e., no one is actively searching for the species), the lag should be accounted for in terms of the detection rate *p*. If, however, the lag has biological causes (e.g. adaptation, novel interactions, climatic change, Allee effects), it can be modelled explicitly by adjusting the functional description of *v*_*t*_ (or *α*_*t*_). As with modification (4), the solution may be a breakpoint model (cf. Aikio et al. 2010) or a gradual increase in *v*_*t*_, which may again be based on the logistic function.
6. Expansion speed does not have to be constant. Whereas Eqs. 6 and 8 assume that *v* is constant and the AOO increases *quadratically* with time, other assumptions may be more realistic for some species. For instance, if the AOO can be assumed to increase approximately *linearly*, this can be modelled by substituting $\alpha_{t} = 1 + \lambda (t - t_{0} )$ in Eq. 4, where *λ* is the average number of new grid cells colonised each year. On the other hand, if the AOO can be assumed to increase approximately *exponentially*, this can be modelled by substituting $\alpha_{t} = e^{{\gamma (t - t_{0} )}}$ in Eq. 4, where *γ* is the annual multiplication rate of occupied grid cells. Other forms of increase can be taken into account accordingly by adjusting Eq. 4.
7. The current dark figure *ξ*_*z*_ is fixed at some pre-specified level (*z* being the most recent year in the dataset). In many cases, researchers have a qualified opinion on the dark figures of a species (based on its detectability and the sampling effort). Even though this qualified opinion is not itself estimated numerically, this modification reduces the number of parameters to be estimated, thereby reducing the uncertainty of the remaining parameters, so that the net effect may in fact be more reliable parameter estimates. This modification can be implemented by calculating the start of the expansion process (*t*_0_) from the other parameters. For the assumption of a constant $v = \hat{v}$, for example, the formula becomes
  11 $$t_{0} = z + \sqrt {\frac{{A_{0} }}{\pi }} \cdot \frac{{1 - \sqrt {\xi_{z} \varOmega_{z} } }}{{\hat{v}}}.$$
8. As long as the AOO is increasing, the assumption that occurrences do not disappear (i.e. that grid cells remain colonised) can be relaxed. This modification does not require any changes in the model, but only in the format of the input dataset. (If occurrences can easily disappear, it does not suffice to report the first observation of an occurrence, but the dataset would need to report the presence/absence of each occurrence for each time step thereafter). The model does not care about the identity of occurrences, only about their number. Provided that the net change in AOO is positive, the model will remain applicable.
9. Grid cells are not squares with 2 km edge length. This convention was merely chosen to comply with IUCN’s (2017) recommendations. However, the value assigned to *A*_0_ does not have to be 4 km^2^, nor do grid cells have to be squares. For instance, Breiner and Bergamini’s (2018) suggestion to use a circular buffer approach would be a possible alternative. The only caveat in this regard is that one should not compare expansion speeds of different species that have been estimated using different grid cells.

So far, modifications 1, 2, 7 and 8 are implemented by the ‘expansion’ script (Sandvik 2017). The other modifications are intended to be implemented in future versions.

### Estimating Expansion Speed with Sparse Data

The estimation described above requires a certain amount of observational data, since the time series length should be several times larger than the number of parameters estimated. This amount of data will not always be available, however. An alternative estimation method that requires fewer years of data is possible when expressing expansion speed as

12 $$\hat{v} = \frac{{\sqrt {A_{t} } - \sqrt {A_{t - \tau } } }}{\tau \sqrt \pi },$$

where *A*_*t*_ is defined as above (the AOO in year *t*); and *τ* is the number of years between the two AOO estimates. This follows directly from the definition of expansion speed (cf. Eq. 7).

This method does not account for detection rate or dark figures. However, in the absence of sufficient data, it provides a straightforward, if rough, estimate of the expansion speed. By multiplying *A*_*t*_ and *A*_*t*–τ_ by *ξ*_*t*_, dark figures may be included into the estimate based on expert judgement alone. Since this method is only recommended when too few years of data exist, *τ* will normally be small, so that the change of the dark figure during *τ* is negligible.

## Test of the Model

### Simulations

The model was tested using 1000 simulated expansion and detection processes. The radius of the AOO was assumed to increase linearly over time. Therefore, the simulations did not test the *assumptions* of the model (which would have to be investigated empirically), but the *efficacy* of the model to produce robust estimates of *v*, *p* and *t*_0_. In each simulation, the expansion speed, the start of the expansion and the detection rate were determined as *v* = 20 · 100^*U*^, *t*_0_ = 1900 + 100 *· U*, and *p* = 0.002 · 100^*U*^, respectively, where *U* is a uniformly distributed random variable within the bounds ]0;1[. For each year between *t*_0_ and *t* = 2018, a number of observations was drawn from a binomial distribution, *ω*_*t*_ ~ *B*(*φ*, *p*), as defined above. This set of observations was used as input to the model, which estimated $\hat{v}$, $\hat{t}_{0}$ and $\hat{p}$ from the data. The estimates could then be compared to the true values for these three variables and the dark figure *ξ*_*z*_ in the last year.

The model was run both in its maximum-likelihood version (Eq. 9) and its least-squares version. In separate model runs, the dark figure was fixed at the true value *ξ*_*z*_ or constrained to intervals [*ξ*_*z*_ · *m*^−1^; *ξ*_*z*_ · *m*], where *m* took the values 1.25, 1.5, 2 and 3. In a final set of model runs, the dark figure was constrained to intervals [0.1 *m*; *m*], where *m* took the values 3, 4, 5, 7.5, 10, 12.5, 15, 20, 25, 30, 40, 50, 75, 100 and 125, excluding intervals that did not contain the true dark figure. The estimation error was calculated as the log-transformed ratio between the median estimate and the true value. Factors affecting estimation error were analysed using linear mixed-effects models (Bates et al. 2015) with simulated datasets as random factors.

### Real-World Data

In addition, the model was tested using real-world data, viz. datasets based on herbarium records on alien plants collected by Norwegian museums and other reported observations, e.g. from the Norwegian Species Observation System (Artsdatabanken et al. 2018). The species included were *Arabidopsis arenosa* (sand rock-cress, 1122 records from 109 years), *Bunias orientalis* (warty-cabbage, 3546 records from 97 years), *Cotoneaster lucidus* (shiny cotoneaster, 308 records from 37 years), *Elodea canadensis* (Canadian waterweed, 142 records from 51 years), *Heracleum persicum* (Persian hogweed, 965 records from 47 years), *Impatiens glandulifera* (Himalayan balsam, 296 records from 48 years), *I.* *parviflora* (small balsam, 175 records from 45 years), *Juncus tenuis* (slender rush, 403 records from 62 years), *Lepidotheca suaveolens* (pineappleweed, 672 records from 109 years), *Rosa rugosa* (beach rose, 3231 records from 64 years), *Salix euxina* (crack willow, 70 records from 34 years) and *Vincetoxicum rossicum* (pale swallowwort, 201 records from 73 years).

For each species, three models were fitted, viz. the default method (Eq. 8) and modifications 1 and 2 (see above). Sampling effort (needed for modification 1) was defined as the total number of herbarium records collected in Norway in each year, i.e. including all flowering plants (both native and alien).

## Results

### Simulations

When dark figures were fixed at the true level, estimates of the remaining parameters were unbiased and very close to the true values. On average, expansion speed was underestimated by 0.2%, detection probability by 0.3%, and the start of the expansion by 1.3 years (Table 2). 95% confidence intervals of the expansion speeds contained the true value in 96% of the simulations.

**Table 2:** Accuracy and precision of expansion parameters estimated from simulated expansion processes (*N* = 1000 simulations), compared to the true parameter values

*m*	Precision		Deviation of estimated from true value			
	*v* < LCI	*v* > UCI	Expansion speed *v*	Detection rate *p*	Start of expansion *t*_0_	Dark figure *ξ*_*z*_
1.00	1.7%	2.4%	− 0.2% (− 13%; + 22%)	− 0.3% (− 32%; + 57%)	− 1.3 a (− 11.7 a; + 12.5 a)	0
1.25	1.7%	0.8%	+ 1.0% (− 13%; + 21%)	− 4.6% (− 60%; + 41%)	− 1.7 a (− 12.9 a; + 10.5 a)	+ 1% (− 2%; + 20%)
1.50	0.9%	0	+ 2.3% (− 13%; + 22%)	− 9.0% (− 70%; + 41%)	− 2.0 a (− 14.2 a; + 9.9 a)	+ 1% (− 2%; + 30%)
2.00	1.1%	0	+ 5.6% (− 13%; + 25%)	− 18.9% (− 79%; + 36%)	− 2.5 a (− 16.0 a; + 9.7 a)	+ 11% (− 2%; + 52%)
3.00	0	0	+ 14.2% (− 13%; + 37%)	− 37.2% (− 86%; + 39%)	− 3.0 a (− 17.8 a; + 9.7 a)	+ 31% (− 13%; + 77%)
3.16	1.7%	0.3%	+ 12.9% (− 38%; + 87%)	− 31.9% (− 82%; + 200%)	− 1.7 a (− 15.4 a; + 14.4 a)	+ 25% (− 61%; + 250%)

Precision is only provided for expansion speed (*v*), in terms of the percentage of simulations whose 95% confidence intervals did not contain the true value (LCI/UCI, lower/upper confidence interval). Accuracy (or lack thereof) is shown in terms of the estimation error, i.e. the deviation of estimated from true values in percent of the true value (estimated value divided by true value minus 1) for expansion speed, detection rate and dark figure; for the start of expansion, the estimation error is provided in absolute terms (estimated year minus true year). The parameter *m* indicates the width of the interval to which dark figures were constrained: for values of *m* from 1 to 3, dark figures were constrained to the interval [*ξ*_*z*_ · *m*^−1^; *ξ*_*z*_ · *m*] centred around the true dark figure *ξ*_*z*_; for *m* = $\sqrt {10}$ ≈ 3.16, intervals contained the true dark figure, but were not centred on it

**Fig. 2:**
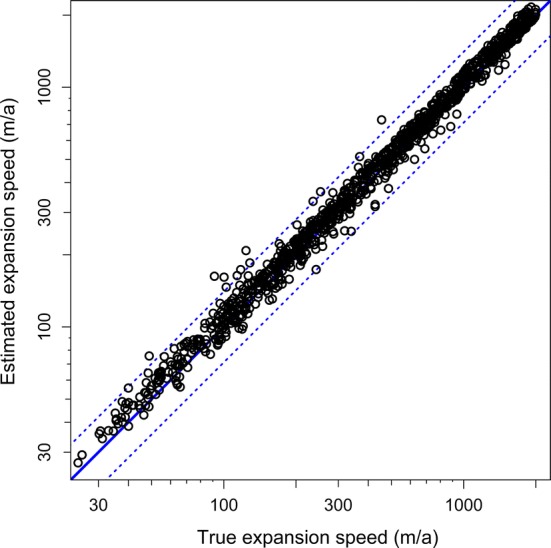
Comparison of true and estimated expansions speeds from the simulations described in the text. The estimates have a high accuracy (weakly biased upwards) and a high precision (the estimated 95% confidence intervals contain the true value in all but 11 cases, *N* = 1000; values are based on a model run in which dark figures were constrained to differ by a factor of maximum 2, upwards and downwards, from the true value). Note that both axes are on log-scale

If dark figures were estimated from the data as well, estimates were somewhat more biased (Fig. 2). If, for example, dark figures were constrained to an interval spanning one order of magnitude (e.g. from 2 to 20), expansion speed was overestimated by 13%, detection probability was underestimated by 32%, the start of the expansion was underestimated by 1.7 years, and the dark figure was overestimated by 25%. Despite these biases, 95% confidence intervals of the expansion speeds contained the true value in 98% of the simulations (Table 2).

The estimation error of expansion speed thus varied very strongly with the width of the constraint interval imposed on the dark figure (*t* = 52.9, ΔAIC = 2111.0). The estimation error also decreased with increasing (true) expansion speed (*t* = –11.3, ΔAIC = 117.6) and with increasing length of the observational time series (*t* = 4.4, ΔAIC = 17.7; *N* = 4916 models on 1000 datasets).

All values reported above were obtained by using the least-squares method. Estimates obtained using the maximum-likelihood method were both less accurate (more biased) and less precise than the former, especially when dark figures were not constrained to narrow intervals (results not shown).

### Real-World Data

**Fig. 3:**
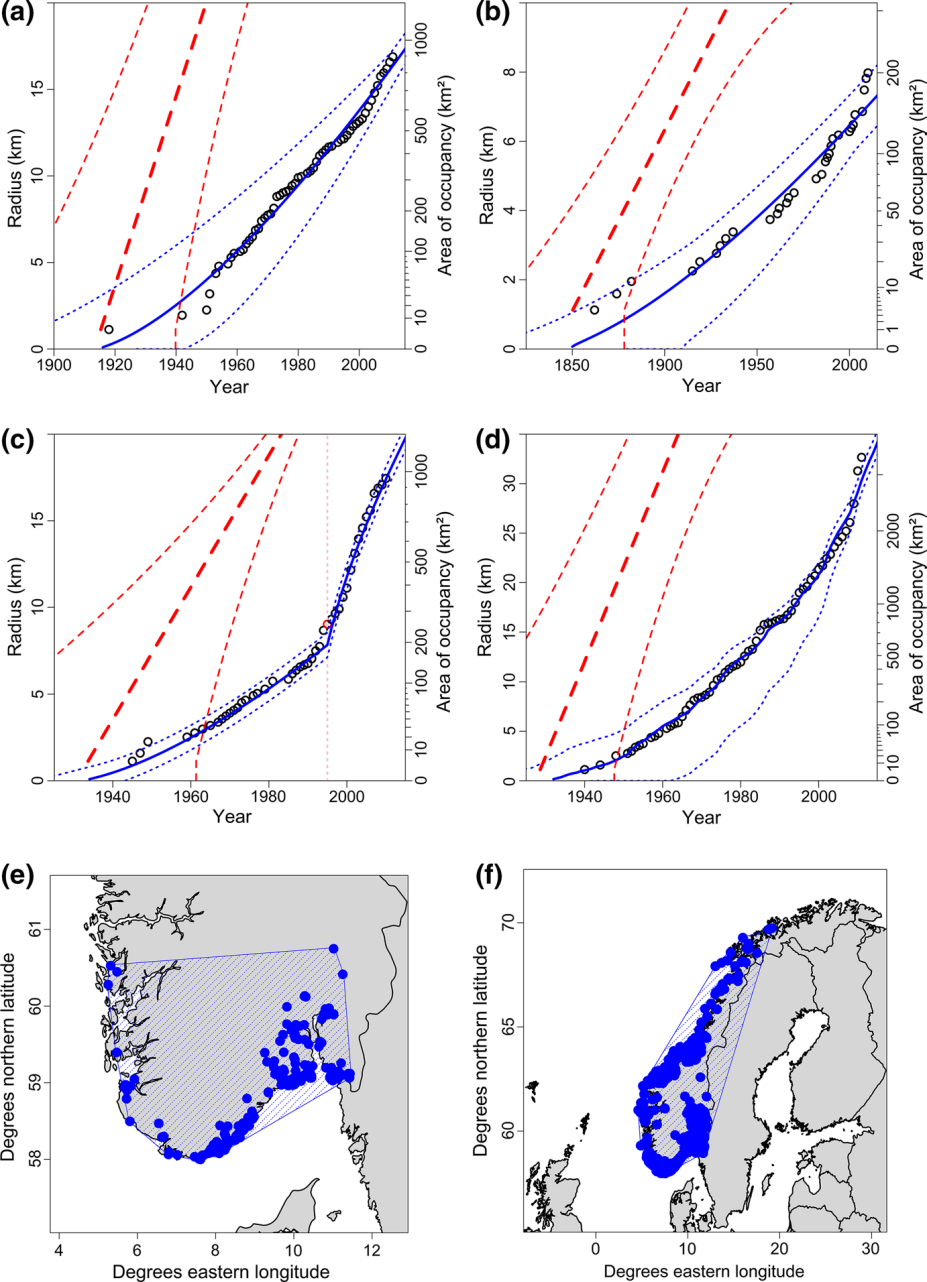
Application of the expansion model to observational data on four alien plant species in Norway. Expansion trajectories of **a***Juncus tenuis* (slender rush), **b***Salix euxina* (crack willow), **c***Impatiens glandulifera* (Himalayan balsam, model fitted using modification 2, see text), **d***Rosa rugosa* (beach rose, model fitted using modification 1, see text); circles are observation data (known AOO on the right *y*-axes), solid lines represent the best fit to observation data, broken lines represent the estimated total AOOs (*A*_*t*_ on the right *y*-axes, *r*_*t*_ on the left *y*-axes), dotted lines are confidence intervals. Distribution maps of **e***J.* *tenuis* and **f***R.* *rugosa* in Norway; circles are observations, hatched polygons are the known EOOs

Estimates were obtained for the twelve alien plant species (Table 3, Fig. 3). For most species, the standard model was best supported in terms of AIC_C_. For three species, the model with one change in detection rate (modification 2) was better supported than the standard model (Table 3, Fig. 3c). For one species (*R. rugosa*), the model in which detection rates varied in parallel to overall collection effort (modification 1) was best supported (Fig. 3d; $\hat{v}$  = 939 m/a [95% confidence intervals: 508 m/a; 1107 m/a], $\hat{\kappa }$ = 0.018 [0.008; 0.077], $\hat{t}_{0}$  = 1928 [1904; 1947], *R*^2^ = 0.994, AIC_C_ = 377.2; results are not shown for other species).

**Table 3:** Estimates of expansion parameters for twelve alien plant species in Norway

Species	Known		Estimates (median and 95% confidence interval)				*R*^2^	AIC_C_	
	AOO (km^2^)	EOO (km^2^)	Expansion speed *v* (m/a)	Detection rate *p* (‰)	Start of expansion *t*_0_	Dark figure *ξ*_*z*_		one *p*	two *p*
*Arabidopsis arenosa*	2728	571,000	564 (294; 761)	3.8 (1.8; 15.4)	1882 (1857; 1898)	6 (2; 10) [5]	0.974	**614.8**	615.1
*Bunias orientalis*	1496	118,000	299 (171; 450)	1.6 (0.6; 6.2)^a^	1847 (1804; 1876)	5 (2; 10) [5]	0.988	431.0	**427.7**
*Cotoneaster lucidus*	924	322,000	911 (413; 1416)	12.1 (4.8; 59.0)	1966 (1944; 1979)	6 (2; 10) [5]	0.938	**195.2**	195.3
*Elodea canadensis*	328	184,000	212 (124; 337)	5.2 (2.3; 18.5)	1913 (1885; 1936)	5 (2; 8) [4]	0.934	**136.5**	137.4
*Heracleum persicum*	1776	603,000	578 (254; 1003)	0.8 (0.2; 3.5)^b^	1907 (1844; 1947)	7 (2; 10) [5]	0.965	232.2	**224.9**
*Impatiens glandulifera*	956	387,000	490 (246; 736)	4.1 (1.5; 18.5)^c^	1937 (1900; 1962)	4 (2; 6) [3]	0.994	232.2	**231.0**
*Impatiens parviflora*	376	73,100	405 (262; 666)	2.8 (1.1; 7.3)	1927 (1895; 1948)	10 (5; 20) [10]	0.940	**142.9**	143.6
*Juncus tenuis*	896	84,300	543 (346; 912)	3.1 (1.2; 7.8)	1915 (1882; 1940)	10 (5; 20) [10]	0.986	**271.9**	273.9
*Lepidotheca suaveolens*	2308	681,000	482 (273; 679)	4.2 (1.8; 2.1)	1875 (1853; 1896)	6 (2; 10) [5]	0.976	**629.3**	630.3
*Rosa rugosa*	3352	397,000	1090 (620; 1600)	5.4 (2.4; 23.1)	1939 (1917; 1954)	6 (2; 10) [5]	0.985	**377.7**	379.1
*Salix euxina*	200	27,100	102 (64; 150)	3.1 (1.4; 11.0)	1850 (1808; 1878)	5 (2; 8) [4]	0.943	**104.6**	106.1
*Vincetoxicum rossicum*	96	1700	39.0 (23.7; 64.3)	7.2 (2.2; 298.8)	1822 (1792; 1862)	3 (1; 6) [3]	0.955	**56.2**	—^d^

Parameters given are the known area of occupancy (AOO), extent of occurrence (EOO, not corrected for coastline or political borders); estimates of expansion speed, detection rate, start year of the expansion, dark figure in the last year of the dataset; variance explained (*R*^2^); and Akaike’s Information criterion (corrected for small sample size, AIC_C_) for two different models (‘one *p*’, detection rate is constant; ‘two *p*’, detection rate is allowed to change once). Estimates are based on the model with the lowest AIC_C_, which is emphasised using boldface. Parentheses contain 95% confidence intervals. Square brackets contain the pre-specified dark figure *ξ*_*z*_ (meaning that dark figures were constrained to the interval [10^−0.5^ *ξ*_*z*_; 10^+0.5^ *ξ*_*z*_])

^a^Detection rates after 2000 were 12.8‰ (4.3‰; 52.7‰)

^b^Detection rates after 2003 were 13.9‰ (5.6‰; 65.6‰)

^c^Detection rates after 1994 were 18.5‰ (8.7‰; 61.0‰)

^d^The dataset contained too few years with unique observations (*N* = 21) to fit a model with two detection rates

Expansion speeds for the different species varied between 39 and 1100 metres per year. Detection rates varied between 0.0008 and 0.0185 (Table 3). In terms of the variance explained, all models obtained a very good fit (all *R*^2^ > 0.93; cf. Fig. 3).

## Discussion

Expansion speed—understood as the increase in the radius of a coherent circle having the same area as the AOO (area of occupancy)—is here suggested as a descriptive measure of the ability of an alien species to increase its spatial presence in an assessment area (see also Hill et al. 2001; Catford et al. 2016). Expansion speed represents one dimension of invasiveness, as AOO is one of the factors determining the impact of alien species on native biota (in addition to the local densities obtained and the per-capita ecological effects, Parker et al. 1999). Some impact assessment schemes of alien species explicitly incorporate spatial criteria (e.g. Essl et al. 2011; D’hont et al. 2015). Expansion speed, as defined in this paper, is currently used as one of three criteria describing invasion potential in the Generic Ecological Impact Assessment of Alien Species (GEIAA, Sandvik et al. 2019). This method is used to classify alien species in Norway and Sweden according to their environmental impact on native biota (Artsdatabanken 2018; Strand et al. 2018).

Some aspects of the expansion speed deserve further discussion, viz. its definition, its underlying assumptions, its measurement unit, and its estimation. This is done in the following paragraphs.

### Definition

Expansion speed, as defined here and by some earlier authors (Hill et al. 2001), represents the increase in AOO, irrespective of the mechanisms or pathways involved. In addition to natural dispersal (active or passive), this includes human transport within the assessment area (intentional or unintentional), and even novel anthropogenic introductions *into* the assessment area. It might, at first sight, seem counterintuitive to lump together natural and anthropogenic factors into a single measure. However, the invasiveness of many alien species is mainly due to these anthropogenic factors. *Arion vulgaris* (Spanish slug) is a case in point: its expansion is not so much due to the high dispersal velocity of this gastropod, but rather due to its close association to horticultural products, which are transported over huge distances (Zemanova et al. 2016).

Being defined in terms of AOO, expansion speed should not be envisaged as an equivalent to dispersal velocity, to the velocity of an actual invasion front, or to the rate of range expansion. Only under highly unlikely conditions would these measures be identical, viz. if there is exactly one centre of spread (one source population) and if no grid cells behind the invasion front remain unoccupied. For example, in Fig. 3e, f, only minor parts of the species’ EOOs or ranges (the hatched polygons) are actually occupied (filled with circles). It is thus evident that expansion speed measures a different aspect of an invasion process than do other metrics, such as dispersal velocity.

Expansion speed is intended to quantify the increase in the spatial component of impact, which is closely related to AOO, but not necessarily to EOO, range or dispersal distance. As such, expansion speed is neither intended as a replacement of dispersal velocity, nor can it be inferred directly from estimates of dispersal velocity. On the other hand, the estimation of expansion speed is possible even in cases where the dispersal velocity is unknown, since all that is needed is a dataset with the time and place of observations. Such occupancy data are available from a number of sources (e.g. natural history collections in museums, national record databases, monitoring programs).

### Expansion Pattern

The model presented (Eq. 4) is valid for all kinds of expansion patterns. However, for its parameters to be estimable, one is bound to make some simplifying assumptions. The simplest set of assumptions—constant expansion speed and detection rate—requires the radius of the AOO to increase linearly, and thus AOO to increase quadratically over time (see Eq. 8 and Fig. 1). An assumption of quadratic increase in AOO is justified in several situations, e.g. whenever a species expands with an approximately bivariate-Gaussian dispersal kernel (e.g., McGeoch and Latombe 2016), independent of the number of source populations and of the proportion of grid cells occupied behind the invasion front.

However, other situations may warrant other functions, i.e. AOO may increase slower or faster than quadratically. For instance, AOO would increase linearly when a habitat specialist expands due to natural dispersal along linear habitats such as rivers, forest edges or coast lines. As an anthropogenic example of an approximately linear expansion one might think of a species that spreads minimally following introduction, so that all expansion is due to novel introductions (given a roughly constant propagule pressure).

In situations where most spread is due to long-distance dispersal (cf. Nathan et al. 2002; Katul et al. 2005), however, AOO would increase approximately exponentially and thus faster than quadratically. This would be the case for distributions whose mode is sufficiently far from the source location (such as a gamma distribution). Fat-tailed or stratified dispersal functions (cf. McGeoch and Latombe 2016) would be intermediate to the quadratic and the exponential situation.

Given that the increase of AOO is known or assumed to be non-quadratic, Eq. 4 would have to be modified accordingly (see modification 6 above). Several factors make it likely that even seemingly non-quadratic expansions can be approximated by the quadratic model (Eq. 8), however. First, even many linear habitats, such as rivers, can branch. Second, dispersal or anthropogenic transport may happen from one linear habitat plot, e.g. a forest edge, to another. In cases of long-distance dispersal, the increase in AOO is exponential only under the boundary condition that grid cells are very unlikely to be colonised from different source cells. In most realistic situations, this will not be the case, and the increase in AOO would be somewhat intermediate to the quadratic and the exponential model. It will differ from case to case whether the increase in AOO can be approximated to be quadratic. In principle, and given sufficient data, this can be tested by fitting different models to the set of observations and comparing the resulting AIC_C_ values.

### From Which Part of the Expansion Process Should the Speed be Estimated?

If the increase of AOO cannot be approximated as quadratic, expansion speed is no longer constant during the invasion process. Cases in which AOO increases linearly or exponentially have just been described (cf. modification 6); in addition, there may be lag phases at the beginning of an expansion (Crooks 2005; Aikio et al. 2010; Coutts et al. 2018) and/or a deceleration of expansion speed when a species approaches full colonisation of its potential AOO (cf. modifications 4 and 5).

In all these cases, the expansion speed depends on when during the expansion process it is estimated. As a means of standardisation, it is suggested to report the *highest realistic* value of expansion speed that is measured, estimated or reported. This choice can be rationalised as following directly from the aim of expressing expansion potential as a *propensity*, and independently from contingent facts such as when the species had been introduced. If the average expansion speed was to be used, an initial lag phase would reduce the estimate. If the current expansion speed was to be used, a species that has (almost) completed its expansion, has no (or a low) expansion; as soon as the species is removed from parts of its AOO, however, it will again have the ability to expand much faster. This is why expansion speed ought to capture the *highest* potential expansion ability.

When only few years of data are available, a high estimate of expansion speed may be due to observation error, measurement error or very special (and non-representative) conditions. For this reason, the use of an upper confidence limit (e.g. the 75th or 95th percentile) may be more *realistic* than strictly reporting the maximum value. Furthermore, if the cause of high expansion speed in the past has ceased to exist and is very unlikely to return, estimates should not be based on the historical increase in AOO. For example, many terrestrial plant seeds were spread with the ballast soil of (sailing) vessels. Ballast soil having been replaced by ballast water, the former pathway is not relevant any more.

### Measurement Unit

According to the definition proposed here, expansion speed is the annual increase in the *radius* of the AOO, thus measured in metres per year (m/a). In principle, different ways to measure, or units in which to express, expansion speed might have been possible, e.g. (i) as an *absolute* increase in AOO, measured in km^2^/a; (ii) as an increase in AOO *relative to the AOO at the time of assessment* (‘current AOO’), measured in percent per year; or (iii) as an increase in AOO *relative to the area that might potentially get colonised* (‘final AOO’), measured in percent per year. The disadvantage with such measurement units is that expansion speed would become a function of time: the absolute increase in AOO, as well as the increase relative to the final AOO, is *positively* related to the duration of the expansion; the increase relative to the current AOO is *negatively* related to the duration of the expansion.

Having said this, knowledge of current and future areas constitutes relevant information that ought to be made accessible to biodiversity management authorities. However, this is best accomplished in the form of separate estimates, rather than by trying to incorporate several measures into one estimate of expansion speed.

### Estimability

Estimates of AOO are always uncertain, because detection rates of occurrences hardly ever reach 100%. Detection rates are a function of sampling effort as well as biological characteristics of the species (its size, habitat, behaviour etc.). The ratio between the total AOO (observed plus unobserved occurrences) and the known AOO (observed occurrences only) is the dark figure of AOO. The dark figure is by definition unknown (unless it equals 1), but it may be estimated using appropriate sampling designs (see, e.g., Wintle et al. 2004; Stanley and Royle 2005; Christy et al. 2010; McCarthy et al. 2013; Kellner and Swihart 2014).

This uncertainty in AOO affects expansion speed, too (Preuss et al. 2014). Since the uncertainty in AOO is directly proportional to the dark figure, uncertainty in expansion speed is proportional to the square root of the dark figure. The model presented in this paper allows an estimation of detection rates (and dark figures) alongside the expansion speed and the start of the expansion. However, simulations have shown that detection rates tend to be somewhat underestimated (and, consequently, dark figures to be overestimated). Where expert judgements of the dark figure exist, these can be used to improve the estimates of expansion speed.

Application of the model to a few real-world datasets resulted in good model fit (Fig. 3), although a comparison with the true values of the estimates is of course not possible in this case. Furthermore, all species tested were terrestrial plants. It would thus be useful to test the model with datasets on more species, including a more diverse set of taxa and habitats.

For four of the species, models with variable detection rates were better supported than models with constant detection rates. For three species, there was a breakpoint at which detection rates increased (Table 3, Fig. 3c). This may have been due to increased sampling effort, e.g. due to a rise in awareness. For the fourth species (*R. rugosa*), detection rates varied in parallel with the sampling effort for all flowering plants (Fig. 3d). Both findings indicate that estimates can be improved considerably if knowledge about detection rates is made explicit and incorporated into the models.

### Conclusion

Expansion speed has several useful properties. It is a rather intuitive measure of the ability of a species to increase its spatial presence in an assessment area. The latter constitutes an essential part of the impact of a species (Parker et al. 1999). Expansion speed is based on the AOO, which is an established measure in conservation biology (IUCN 2017). It is comparatively easy to estimate, in that it only requires occupancy data, rather than prolonged field studies of dispersal. It is a generic measure that allows a direct comparison of expansion speed across taxa and habitats (Artsdatabanken 2018; Strand et al. 2018). Finally, it is a quantitative measure, a property that increases the testability of impact assessments using expansion speed as a criterion (cf. Mace and Lande 1991).

### Electronic supplementary material

Below is the link to the electronic supplementary material.

Supplementary material 1 (PDF 112 kb) Online Resource 1, authored by F.J.A. Jacobs, contains a proof of the magnitude of the detection debt.

## Appendix

In Sect. 3.2, I stated that the curve of the radius *ρ* of the known AOO starts out with a slope of $b = \sqrt p \cdot v$ (see Fig. 1a). This can be derived under the assumption that the slope does not change after *t*_0_, i.e. assuming that only newly colonised occurrences are regarded as unobserved (since it is the accumulation of unobserved occurrences that leads to the increase in slope over time), i.e. $\varphi (t_{0} + \Delta t) = 0$, so that $\omega (t_{0} + \Delta t) = p \cdot \alpha (t_{0} + \Delta t)$ and $\rho (t_{0} + \Delta t) = \sqrt {p \cdot \alpha (t_{0} + \Delta t) \cdot A_{0} \cdot \pi^{ - 1} }$, where Δ*t* is a time interval not larger than one year (or one time step). The slope can then be expressed as

A1 $$\begin{aligned} b & = \frac{{\rho (t_{0} + \Delta t) - \rho (t_{0} )}}{\Delta t} \\ & = \frac{{\sqrt {p \cdot \alpha (t_{0} + \Delta t) \cdot A_{0} \cdot \pi^{ - 1} } - \sqrt {p \cdot \alpha (t_{0} ) \cdot A_{0} \cdot \pi^{ - 1} } }}{\Delta t} \\ & = \frac{{\sqrt {p \cdot A_{0} \cdot \pi^{ - 1} } \left[ {\sqrt {\alpha (t_{0} + \Delta t)} - \sqrt {\alpha (t_{0} )} } \right]}}{\Delta t} .\\ \end{aligned}$$

With $\sqrt {\alpha (t)} = v \cdot (t - t_{0} ) \cdot \sqrt {\pi \cdot A_{0}^{ - 1} } + 1$ [Eq. 7], this becomes

A2 $$b = \frac{{\sqrt {p \cdot A_{0} \cdot \pi^{ - 1} } \left[ {\left( {v \cdot \Delta t \cdot \sqrt {\pi \cdot A_{0}^{ - 1} } + 1} \right) - 1} \right]}}{\Delta t} = \sqrt p \cdot v .$$
