# Supplementary material for: Expansion Speed as a Generic Measure of Spread for Alien Species
Source: Acta Biotheor. 2019 Sep 28;68(2):227–52. doi: 10.1007/s10441-019-09366-8 (PMC7188734; doi:10.1007/s10441-019-09366-8)
Supplement: Supplementary file 1 — Supplementary material 1 (PDF 112 kb) Online Resource 1, authored by F.J.A. Jacobs, contains a proof of the magnitude of the detection debt. [file 10441_2019_9366_MOESM1_ESM.pdf]

Proof concerning detection debt

by F. J. A. Jacobs

This Electronic Supplementary Material is Online Resource 1 of the article:

Sandvik H (2019) Expansion speed as a generic measure of spread for alien species. Acta Biotheoretica. https://doi.org/10.1007/s10441-019-09366-8

Current affiliation of the author of the article:

Hanno Sandvik, Norwegian Institute for Nature Research (NINA), 7485 Trondheim, Norway; E-mail hanno@evol.no

Affiliation of the author of this Online Resource:

Frans J. A. Jacobs, Institute of Biology Leiden, Leiden University, 2333 BE Leiden, Netherlands; E-mail f.j.a.jacobs@biology.leidenuniv.nl

---

For constant p and v, the detection debt is $\delta = \frac{1}{p} - 1$.

Proof

From $v = \sqrt{\frac{A_0}{\pi}} \frac{\sqrt{\alpha_t} - 1}{t - t_0}$ it follows that $\alpha_t = \left(v \sqrt{\frac{\pi}{A_0}} (t - t_0) + 1 \right)^2$. Then r_t, which equals $\sqrt{\alpha_t A_0 \pi^{-1}}$, satisfies: $r_t = v(t - t_0) + \sqrt{\frac{A_0}{\pi}}$.

ρ_t is defined as $\rho_t = \sqrt{\Omega_t A_0 \pi^{-1}} = \sqrt{\frac{A_0}{\pi}} \sqrt{\Omega_t} =$

$$\sqrt{\frac{A_0}{\pi}} \sqrt{p \sum_{i=t_0}^{t} \left(v(i - t_0) \sqrt{\frac{\pi}{A_0}} + 1 \right)^2 (1-p)^{t-i}} = \sqrt{p} \sqrt{\sum_{i=t_0}^{t} \left(v(i - t_0) + \sqrt{\frac{A_0}{\pi}} \right)^2 (1-p)^{t-i}}.$$

Now change the summation variable i into $t-j$. The expression for ρ then becomes:

$$\rho_t = \sqrt{p}\sqrt{\sum_{j=0}^{t-t_0}\left(v(t-t_0-j)+\sqrt{\frac{A_0}{\pi}}\right)^2(1-p)^j} =$$

$$\sqrt{p}\sqrt{\sum_{j=0}^{t-t_0}\left(v^2((t-t_0)^2-2(t-t_0)j+j^2)+2(t-t_0-j)v\sqrt{\frac{A_0}{\pi}}+\frac{A_0}{\pi}\right)(1-p)^j} =$$

$$\sqrt{p}\sqrt{\left(v(t-t_0)+\sqrt{\frac{A_0}{\pi}}\right)^2\sum_{j=0}^{t-t_0}(1-p)^j-2v\left(v(t-t_0)+\sqrt{\frac{A_0}{\pi}}\right)\sum_{j=0}^{t-t_0}j(1-p)^j+v^2\sum_{j=0}^{t-t_0}j^2(1-p)^j}\ .$$

Now use that (in general, and for $c \neq 1$) $\sum_{j=0}^{t} c^j = \frac{1-c^{t+1}}{1-c}$,

$\sum_{j=0}^{t} jc^j = \frac{c}{(1-c)^2}(1-(1+t)c^t+tc^{t+1})$, and

$$\sum_{j=0}^{t} j^2c^j = \frac{c+c^2-(1+t)^2c^{t+1}+(2t^2+2t-1)c^{t+2}-t^2c^{t+3}}{(1-c)^3}.$$

[The first of these three last summation expressions is a well-known result for geometric series. For the second one of these three write

$$\sum_{j=0}^{t} jc^j = \sum_{j=0}^{t}(j+1-1)c^j = \sum_{j=0}^{t}(j+1)c^j - \sum_{j=0}^{t}c^j = \sum_{j=0}^{t}\frac{d}{dc}c^{j+1} - \sum_{j=0}^{t}c^j =$$

$$\sum_{j=0}^{t}\frac{d}{dc}\left(c\cdot c^j\right)-\sum_{j=0}^{t}c^j = \sum_{j=0}^{t}(c^j+c\frac{d}{dc}c^j)-\sum_{j=0}^{t}c^j = \sum_{j=0}^{t}c\frac{d}{dc}c^j = c\frac{d}{dc}\sum_{j=0}^{t}c^j = c\frac{d}{dc}\left(\frac{1-c^{t+1}}{1-c}\right),$$

and the equality $\sum_{j=0}^{t} jc^j = \frac{c}{(1-c)^2}(1-(1+t)c^t+tc^{t+1})$ follows.

The expression for $\sum_{j=0}^{t} j^2c^j$ follows in a similar way, by using that

$\sum_{j=0}^{t} j^2c^j = \sum_{j=0}^{t}(j+2)(j+1)c^j-(3j+2)c^j$ and $(j+2)(j+1)c^j = \frac{d^2}{dc^2}c^{j+2}$.]

Then $\sum_{j=0}^{t-t_0}(1-p)^j = \frac{1-(1-p)^{t-t_0+1}}{1-(1-p)}$,

$$\sum_{j=0}^{t-t_0} j(1-p)^j = \frac{1-p}{(1-(1-p))^2}(1-(1+t-t_0)(1-p)^{t-t_0}+(t-t_0)(1-p)^{t-t_0+1}),$$

and

$$\sum_{j=0}^{t-t_0} j^2(1-p)^j =$$

$$\frac{1-p+(1-p)^2-(1+t-t_0)^2(1-p)^{t-t_0+1}+(2(t-t_0)^2+2(t-t_0)-1)(1-p)^{t-t_0+2}-(t-t_0)^2(1-p)^{t-t_0+3}}{(1-(1-p))^3},$$

and substituting these expressions into the expression for ρ_t yields that

$$\rho_t = \sqrt{p}\times\sqrt{\begin{array}{l}\left(v(t-t_0)+\sqrt{\frac{A_0}{\pi}}\right)^2\frac{1-(1-p)^{t-t_0+1}}{p}- \\ 2v\left(v(t-t_0)+\sqrt{\frac{A_0}{\pi}}\right)\frac{1-p}{p^2}\left(1-(t-t_0+1)(1-p)^{t-t_0}+(t-t_0)(1-p)^{t-t_0+1}\right)+ \\ v^2\left(\begin{array}{l}1-p+(1-p)^2-(t-t_0+1)^2(1-p)^{t-t_0+1}+ \\ \frac{\left(2(t-t_0)^2+2(t-t_0)-1\right)(1-p)^{t-t_0+2}-(t-t_0)^2(1-p)^{t-t_0+3}}{p^3}\end{array}\right)\end{array}} =$$

$$\sqrt{\begin{array}{l}\left(v(t-t_0)+\sqrt{\frac{A_0}{\pi}}\right)^2 1-(1-p)^{t-t_0+1}- \\ 2v\left(v(t-t_0)+\sqrt{\frac{A_0}{\pi}}\right)\frac{1-p}{p}\left(1-(t-t_0+1)(1-p)^{t-t_0}+(t-t_0)(1-p)^{t-t_0+1}\right)+ \\ v^2\left(\begin{array}{l}1-p+(1-p)^2-(t-t_0+1)^2(1-p)^{t-t_0+1}+ \\ \frac{\left(2(t-t_0)^2+2(t-t_0)-1\right)(1-p)^{t-t_0+2}-(t-t_0)^2(1-p)^{t-t_0+3}}{p^2}\end{array}\right)\end{array}}$$

Now let $t \to \infty$, and use that for $k \geq 0$ $\lim_{t\to\infty} t^k (1-p)^t = 0$. Then

$$\lim_{t\to\infty} \rho_t = \sqrt{\left(v(t-t_0)+\sqrt{\frac{A_0}{\pi}}\right)^2 - 2v\left(v(t-t_0)+\sqrt{\frac{A_0}{\pi}}\right)\frac{1-p}{p} + v^2\left(\frac{1-p+(1-p)^2}{p^2}\right)} =$$

$$\lim_{t\to\infty} \sqrt{\left(v(t-t_0)+\sqrt{\frac{A_0}{\pi}}\right)^2 - 2\left(v(t-t_0)+\sqrt{\frac{A_0}{\pi}}\right)v\frac{1-p}{p} + \left(v\frac{1-p}{p}\right)^2 + \left(\frac{v}{p}\right)^2(1-p)} =$$

$$\lim_{t\to\infty} \sqrt{\left(v\left(t-t_0-\left(\frac{1}{p}-1\right)\right)+\sqrt{\frac{A_0}{\pi}}\right)^2 + \left(\frac{v}{p}\right)^2(1-p)} =$$

$$\lim_{t\to\infty} \sqrt{\left(v\left(t-t_0-\left(\frac{1}{p}-1\right)\right)+\sqrt{\frac{A_0}{\pi}}\right)^2 \left(1+\frac{\left(\frac{v}{p}\right)^2(1-p)}{\left(v\left(t-t_0-\left(\frac{1}{p}-1\right)\right)+\sqrt{\frac{A_0}{\pi}}\right)^2}\right)} =$$

$$= v\left(t-t_0-\left(\frac{1}{p}-1\right)\right)+\sqrt{\frac{A_0}{\pi}}\ .$$

Therefore, in the limit for $t \to \infty$, ρ_t becomes parallel to r_t with a time delay equal to $\frac{1}{p}-1$, which is the detection debt δ.
